# Concurrent imaging of vascularization and metabolism in a mouse model of paraganglioma under anti-angiogenic treatment

**DOI:** 10.7150/thno.40687

**Published:** 2020-02-10

**Authors:** Caterina Facchin, Mailyn Perez-Liva, Anikitos Garofalakis, Thomas Viel, Anais Certain, Daniel Balvay, Thulaciga Yoganathan, Justine Woszczyk, Kelly De Sousa, Joevin Sourdon, Jean Provost, Mickael Tanter, Charlotte Lussey-Lepoutre, Judith Favier, Bertrand Tavitian

**Affiliations:** 1 Université de Paris, PARCC, INSERM, F-75015 Paris, France; 2 BiospaceLab, F-95960 Nesles-la-vallée, France; 3 Engineering physics, Polytechnique Montréal, Montréal, QC, Canada; 4 Montreal Heart Institute, Montreal, QC, Canada; 5 Physics for Medicine Paris (INSERM U1273, ESPCI Paris, PSL University, CNRS FRE 2031), Inserm Technology Research Accelerator for Biomedical Ultrasound, F-75012 Paris, France; 6 Sorbonne Université, Department of nuclear medicine, AP-HP, Pitie-Salpêtrière Hospital, F-75013 Paris, France; 7 Radiology Department, AP-HP Centre, Hôpital Européen Georges Pompidou, F-75015 Paris, France

**Keywords:** Cancer metabolism, angiogenesis, paraganglioma, SDHB, multimodality imaging, positron emission tomography, ultrafast-ultrasound imaging

## Abstract

**Rationale**: Deregulation of metabolism and induction of vascularization are major hallmarks of cancer. Using a new multimodal preclinical imaging instrument, we explored a sequence of events leading to sunitinib-induced resistance in a murine model of paraganglioma (PGL) invalidated for the expression of succinate dehydrogenase subunit B (*Sdhb*^-/-^).

**Methods**: Two groups of *Sdhb*^-/-^ tumors bearing mice were treated with sunitinib (6 weeks) or vehicle (3 weeks). Concurrent Positron Emission Tomography (PET) with 2′ -deoxy-2′-[^18^F]fluoro-D-glucose (FDG), Computed Tomography (CT) and Ultrafast Ultrasound Imaging (UUI) imaging sessions were performed once a week and ex vivo samples were analyzed by western blots and histology.

**Results**: PET-CT-UUI enabled to detect a rapid growth of *Sdhb^-/-^* tumors with increased glycolysis and vascular development. Sunitinib treatment prevented tumor growth, vessel development and reduced FDG uptake at week 1 and 2 (W1-2). Thereafter, imaging revealed tumor escape from sunitinib treatment: FDG uptake in tumors increased at W3, followed by tumor growth and vessel development at W4-5. Perfused vessels were preferentially distributed in the hypermetabolic regions of the tumors and the perfused volume increased during escape from sunitinib treatment. Finally, initial changes in total lesion glycolysis and maximum vessel length at W1 were predictive of resistance to sunitinib.

**Conclusion**: These results demonstrate an adaptive resistance of *Sdhb*^-/-^ tumors to six weeks of sunitinib treatment. Early metabolic changes and delayed vessel architecture changes were detectable and predictable *in vivo* early during anti-angiogenic treatment. Simultaneous metabolic, anatomical and functional imaging can monitor precisely the effects of anti-angiogenic treatment of tumors.

## Introduction

In his seminal paper relating the discovery of a factor activating tumor angiogenesis, Judah Folkman concluded “*that blockade of this factor […] might arrest solid tumors at a tiny diameter of a few millimeters*” 1. The next decades identified the diverse forms of Vascular Endothelial Growth Factors (VEGF) and VEGF-Receptors (VEGFR) as *bona fide* molecular targets for anti-angiogenic therapy of tumors and the first anti-VEGF drug was launched in 2004. Since then, more than ten anti-angiogenic drugs targeting VEGF signaling have been approved by the Food and Drug Administration (FDA) and/or the European Medicines Agency (EMA). Anti-angiogenics are indicated as first and second line drugs, as monotherapy or in association with chemotherapy, for various solid tumors including colorectal cancer, renal cell and hepatocellular carcinomas, thyroid and pancreatic cancer as well as neuroendocrine tumors 2. Highly-vascularized tumors are natural candidates to anti-angiogenic treatments, which include, aside renal carcinoma 3, the paraganglioma/pheochromocytoma (PPGL) cluster of neural-crest derived tumors that arise in the sympathetic and/or parasympathetic nervous system, and in the chromaffin cells of the adrenal medulla, respectively 4.

Fifteen years after their introduction in clinical practice, anti-angiogenics represent a small niche in the cancer pharmacopeia. Although the concept forged by Folkman 50 years ago appears to stand on solid scientific grounds, clinical benefits are often limited, with a significant level of toxicity and frequent escape from treatment 2,5. Two phase II randomized clinical trials of sunitinib, a multi-tyrosine kinase inhibitor (TKI) that targets VEGFRs and inhibits cell proliferation 6, are ongoing in Europe (FIRSTMAPP) and Canada (SNIPP) 7,8, in patients with malignant PPGL. Regarding PPGL, intermediate results combining the SNIPP trial and a review of the literature indicate a positive response to sunitinib in 72% of patients, among these 62% showing a stable disease and 35% a partial response 9,10. Complete response was only achieved in one reported case 11. These studies have suggested that SDH-mutated patients have better response than non-mutated patients. Although the duration of follow up was highly variable (from 1 to 88 months), acquired resistance was described in a substantial number of patients (8/26 responders in 10). The modest clinical efficacy of anti-angiogenics remains poorly understood 2,12, as we lack reliable biomarkers predictive of response 2,12,13. Interestingly, recent studies in preclinical rodent models and in patients suggested that escape from sunitinib treatment could be caused by a switch of the tumor metabolism rather than by acquired insensitivity to VEGF blockage 14-16.

We reasoned that *in vivo* concurrent determination of tumor metabolism and vascularization 17 by noninvasive imaging could provide new insights into the mechanism of tumor escape from sunitinib treatment. Here, we report the response to sunitinib treatment in a murine model of PPGL using a new trimodal imaging instrument, PETRUS 18, that combines anatomical imaging with X-ray computed tomography (CT), metabolic imaging with positron emission tomography (PET) with 2′ -deoxy-2′-[^18^F]fluoro-D-glucose (FDG) and vascular imaging using Ultrafast Ultrasound Imaging (UUI). We describe the sequence of events that lead the tumors to resume growth under sunitinib treatment, and show that noninvasive imaging may provide early predictors of escape.

## Material and Methods

### General description and protocols

**Figure 1:**
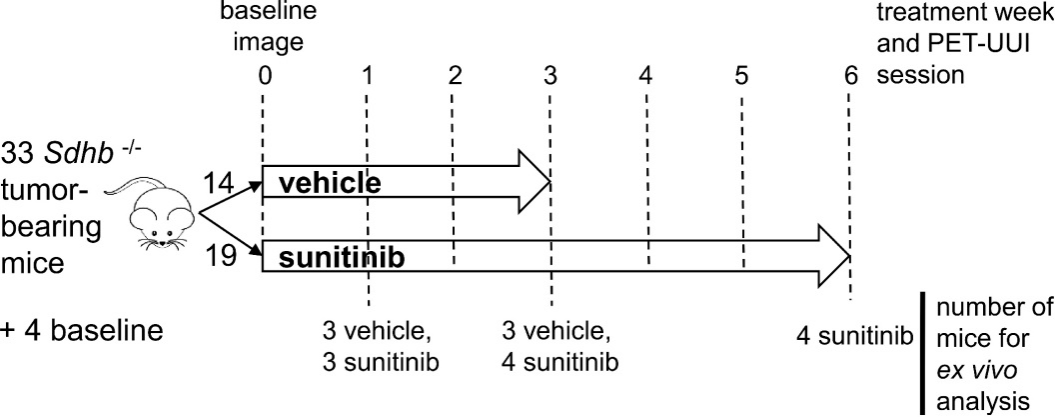
** Study design.** Mice were divided into two groups: sunitinib- and vehicle-treated (SUNI and VEH, respectively). 8 mice from each group, were scanned with PETRUS before and after 1, 2 and 3 weeks of treatment. In addition, SUNI mice were imaged at 4, 5 and 6 weeks of treatment. Among the two groups, n=21 Sdhb^-/-^ tumor bearing mice were used for histological and western blots analysis at baseline (no treatment, n=4), week 1 (SUNI, n=3; VEH, n=3), week 3 (SUNI, n=4; VEH, n=3), week 6 (SUNI, n=4).

Animal experiments were approved by the French Ethical committee under reference No 16-098 and performed by certified personal following the French law on animal experimentation n°2013-118. Allografts of tumors obtained from immortalized mouse chromaffin cells (imCC) carrying a homozygous knockout of the *Sdhb* gene (*Sdhb^-/^*^-^) were propagated in the back of the neck fat pad of NMR nude female mice (n= 37, 6 weeks old, weight= 30g, Janvier Labs, France). Mice were maintained in controlled temperature (24°C) and relative humidity (50%) on a 12/12-light/dark cycle with free access to water and food*.* The tumor volume was calculated by daily caliper measurements using the formula: ½ x length × (width)^2^. Once the tumour volume reached 140 mm³, mice with *Sdhb*^-/-^ tumors were randomly divided into two groups (**Figure 1**), one treated with sunitinib and the other with vehicle. The sunitinib group (SUNI, n=19) received sunitinib malate (Cliniscience, A10880-500), at a dose of 50mg/kg body weight during 6 consecutive weeks, administered daily by oral gavage of 200μL of a 10mg/mL DMSO/PBS (1:4) solution. The vehicle group (VEH, n=14) received 200 µL of the DMSO-PBS solution (1:4) daily during 3 weeks, after which the tumor volume exceeded UKCCCR recommendations and mice started to show signs of advanced cancer disease 19 and were humanely euthanized.

### In vivo experiments

16 mice (n=8 SUNI and n=8 VEH) were used to monitor the effect of sunitinib using PETRUS (PET Registered Ultrafast Sonography; 18), a new hybrid imaging instrument based on simultaneously acquired PET/CT and UUI. A custom-made ultralight probe (Vermon, France) with 15 MHz central frequency, 128 transducer elements and 100μm pitch was attached to the UUI scanner as in 20. The probe scanned the whole tumor using a high-precision micropositioner (Hexapod H811, Physik Instrumente, Germany; minimum incremental motion 0.2 μm) during PET acquisition, as in 18.

The timeline for *in vivo* imaging is depicted in **Figure 1**. All animals underwent a baseline imaging session the day preceding the first administration of drug or vehicle. Imaging was repeated periodically every week during 6 weeks for the SUNI group and during 3 weeks for the VEH group (**Figure 1**).

Prior to each imaging session, mice were fasted overnight and anesthetized with isoflurane 2.0±1.0 % (IsoVet 100%; Centravet, France) in 100% O_2_. Body weight and caliper tumor volumes were measured every day and glycaemia once a week before the PET-acquisition. A home-made catheter with a 25G needle (Fischer Scientific, France) connected to a 5cm polyethylene tubing (Tygon Microbore Tubing, 0.010" x 0.030"OD; Fisher Scientific, France) was inserted in the caudal vein for radiotracer injection. Mice were then placed head-first in supine position in the bed of the PET/CT scan under controlled respiration rate (60 to 80 cycles per minute) and controlled body temperature of 37°C throughout the entire imaging session.

To select the volume to image with UUI, the tumor margins were localized using B-mode image with the micro-positioner robot and stored. An X-ray CT scan was acquired in semi-circular mode, 39kV tension, 720 projections full scan, 300ms per projection, and a binning 1:4. CT data was reconstructed using filtered back projection (filter: Cosine; Cutoff: 100%) 21, with a pixel size and slice thickness of 0.23 mm. The PET acquisition was set to start 30 seconds before injection of 10 MBq of FDG in 0.2 mL saline into the tail vein of the mice. List-mode PET data were collected during 60.5 min, binned using a 5-ns time window, a 400- to 600-keV energy window, and a 1:5 coincidence mode. Attenuation correction was based on the X-ray CT image with the ultrasound probe in place over the tumor volume. A dynamic sequence composed of 31-frames with the following time sequence: exclusion of 20 s; 10 frames of 5 s, 5 frames of 10 s, 2 frames of 15 s, 3 frames of 60 s, 5 frames of 120 s, 3 frames of 5 min, 3 frames of 10 min. Reconstruction used Tera-Tomo® (3D-OSEM based algorithm, Mediso, Hungary) with expectation maximization iterations, scatter and attenuation correction. Ultrafast Doppler images were obtained using dedicated Matlab® (The MathWorks, Natick, MA, USA) scripts to perform the beamforming of plane waves both in transmission and reception. Doppler was started 5-10 minutes after FDG injection and, depending on the size of the tumor, continued over 20 to 40 minutes during the PET acquisition. Adjacent 2D planes (12.8 x 20 mm^2^) of Ultrafast Ultrasound Doppler were acquired every 0.1 mm over the whole tumor volume. Depending on the size of the tumor, we acquired 70 to 200 planes, each plane being composed of 300 frames acquired at a rate of 500 frames per second by coherently compounding the images of 11 tilted plane waves equally spaced between -10 and 10 degrees. To minimize motion artifacts, image acquisitions were triggered during the respiratory pause of the animal.

For UUI reconstructions, each stack of 300 frames was processed to generate an Ultrafast Power Doppler volume representing the vascular anatomy. Specifically, a spatiotemporal filter based on the singular value decomposition was applied to separate tissue from blood signal, and the power (i.e., the square of the signal amplitude) was then integrated over the 300 frames 22. The process was repeated for each slice and provided a 3D volume of the vasculature of the tumor with an anisotropic resolution that depends on the elevation focusing capability of the ultrasonic probe. To correct for this effect, we de-convolved the 3D volume with a kernel of blurring estimated by the blind deconvolution method 23 applied to a B-mode 3D volume of crossed 80-μm copper wires immersed in water. The blurring kernel was then used in a Lucy-Richardson deconvolution 24,25 to establish an isotropy spatial resolution of approximately 100 µm^3^.

### Image and data processing

Each PETRUS imaging session produced a set of registered 3D volumes of CT, dynamic PET and Ultrafast Doppler. Tumor volumes were defined on the CT images. A first volume of interest (VOI) covering the tumor was segmented from the PET images as the voxels with a Standard Uptake Value (SUV) of FDG at 50-60 min post-injection higher than 30% of the peak SUV in the tumor 26. This VOI served as a mask to crop the UUI volume acquired simultaneously in order to define a unique PET-UUI VOI for each tumor, from which all quantitative PET parameters were further derived. The blood input function was derived for each dynamic PET by delineating a VOI over the *vena cava* 27 with the same threshold as for the tumor, based on 30% of peak uptake defined as the five hottest pixel cluster in the VOI 28. PET dynamic analysis was performed by a 3-compartimental model with Patlak linearization calculated using the General Kinetic Modeling Tool program (PKIN) (PMOD Technologies Ltd, Zürich, Switzerland). The Metabolic Rate of Glucose (MRGlu) consumption, for accurate quantification of FDG consumption, was expressed as: MRGlu = Ki×PG/LC, where Ki = (K1×k3)/(k2+k3) is the metabolic flux based on rate constants of FDG transport from the vessel to the cell cytoplasm, PG is the plasma glucose concentration and LC is the lumped constant. Here, we fixed LC at a value of 0.69 based on 29,30. The 3 rates constants describe respectively: the transport from the plasma to the first tissue compartment (K1), the reverse transport from the first tissue compartment to plasma (k2) and the transport from the first tissue compartment to the second tissue compartment, in which FDG is phosphorylated to FDG-6-P (k3). Accordingly, MRGlu reflects both vessel permeability and the phosphorylation of FDG. The vascular network of the tumor was analyzed by post-processing of the UUI Doppler acquisition. First, the vessels were segmented from the background using a classical Hessian-based vessel enhancement method 31,32. Next, the filtered image was segmented using an isodata unsupervised classification method 33. Vessels were then skeletonized using an iterative ordered thinning-based skeletonization method 34,35 for topological analysis of their shape parameters. Finally, the skeletonized structure was converted into a network graph described by nodes and edges 36,37. From the processed vessels network, we analysed the mean, minimum and maximum vessels length and radius, mean vessel tortuosity (shortest distance between nodes/ vessel length), number of nodes and ultrasound volume (number of voxels comprising the vessel network × voxel volume), using standard MATLAB® routines. Finally, each PET-UUI volume was segmented into three sub-regions based on SUV values, i.e. SUV 1-2, SUV 2-3, SUV >3, and UUI-derived parameters were calculated in the PET-segmented sub-regions.

### Ex-vivo experiments

A total of 21 SUNI and VEH mice were used for histology and western blots: (i) 4 mice were sacrificed prior to treatment (baseline), (ii) 6 mice (3 SUNI and 3 VEH) were sacrificed after 1 week of treatment, (iii) 7 mice (4 SUNI and 3 VEH) sacrificed after 3 weeks of treatment, and (iv), 4 SUNI mice were sacrificed after 6 weeks of treatment (**Figure 1**). Additionally, at the end of the imaging sessions, tumors were resected and processed for ex-vivo analysis.

### Immunofluorescence

Tumors were fixed overnight in 4% paraformaldehyde, transferred to 70% EtOH and paraffin-embedded. Sections 4µm-thick were deparaffinized, rehydrated and incubated with anti-CD31 (1:100, DIA 310, Clinisciences) followed by goat-anti rat 594 (10348312, Life Technology) and/or with anti- alpha-smooth muscle actin (α-SMA)-Cy3 (1: 250, C6198, Sigma) antibody at room temperature during 1 hour.

Alternatively, sections were incubated successively with GLUT1 (1:1000, ab115730, Abcam) and anti-CD31 (1:600 DIA 310, Clinisciences) using the OPAL 4 color kit (NEL820001KT, PerkinElmer) at room temperature during 1 hour. The secondary antibody was Opal Polymer anti-rabbit HRP (ARR1001KT, PerkinElmer) for GLUT1 and anti-rat HRP (MP-7444-15, Eurobio) for CD31.

Nuclei were stained with DAPI and slides were coverslipped and scanned with Vectra^®^ Polaris^™^ (PerkinElmer). Analysis of immunofluorescence was performed with homemade software developed in Matlab® (The MathWorks, Natick, MA, USA). CD31, GLUT1 and α-SMA signals were counted in 20 fields of 3 independent sections at x20 magnification for each sample. Pericyte coverage was calculated as the number of vessel covered by α-SMA (both α-SMA and CD31 positives vessels) over the total number of CD31 positive vessels.

### Western Blots

Tumor samples were placed in lysis buffer containing Tris-HCl (pH 8.5) and 4% SDS 4 and sonicated. After centrifugation, the proteins in the supernatant were quantified, loaded onto a 10% SDS-PAGE gels (mini-protean TGX gels, BioRad) and transferred to nitrocellulose membranes. The membranes were blocked with 5% milk in PBS and immunoblotted with GLUT1 antibody (1:10000, ab115730, Abcam). Membranes were then incubated with horseradish peroxidase (HRP)-conjugated anti-rabbit secondary antibodies (1:10000, 474-1506, KPL). Chemiluminescence detection was performed using the ECL kit (Clarity Western ECl substrate; BioRad) or SuperSignal West Femto (34094, ThermoScientific). Quantification of immunoblots was done on digitalized images using ImageLab Software 6.01 (BioRad). The intensity of immunoreactive bands was normalized by the loading control (β-tubulin, 1:2000, 802001, BioLegend).

### RNA isolation and qRT-PCR

Total RNA were extracted from formalin-fixed paraffin-embedded (FFPE) sections using AllPrep DNA/RNA FFPE kit (Qiagen, 80234) for quantitative real-time RT-PCR (qPCR). Based on the GLUT1 immunofluorescence signal (IFS), the areas of interest were delineated and isolated for RNA extraction by scraping unstained FFPE sections guided by the GLUT1 IFS using a scalpel under a Wild Heerbrugg microscope. RT was performed using random primers and iScript enzyme (BioRad, 1708891), during 30 min at 42°C and qRT-PCR was performed with SuperScript SybrGreen (BioRad, 1725124). The samples were run in duplicate and two different housekeeping genes (Ubc and B2m) were used for normalization and internal control. Relative gene expression was calculated using the ΔΔCt method. Ubc: F 5′-AGCCCAGTGTTACCACCAAG-3′; R 5′-ACCCAAGAACAAGCACAAG-3′; B2m: F 5'-ATTCACCCCCACTGAGACTG-3'; R 5'-TGCTATTTCTTTCTGCGTGC-3'; VEGF-A: QuantiTect (CA, USA) Primer Assay QT00160769.

### Statistical analysis

Statistical analysis was done using GraphPad Prism (GraphPad Software, San Diego, California, USA). Data are expressed as mean ± SEM. Parameters derived from PET and UUI and histological markers were compared between the tumors of the SUNI and VEH groups using two-way ANOVA with Bonferroni correction. Student's *t* test was used to compare pre-treatment parameters: paired *t*-test for continuous variables after d'Agostino and Pearson's normality test, or using a nonparametric Wilcoxon matched-pairs test if the distribution was not normal. Reported *p* values were two-sided and considered significant when *p* < 0.05.

A heatmap plot was constructed using the square of Pearson correlations (R^2^) between PET-CT-UUI parameters calculated by MatLab® software in n = 12 mice. R^2^ with p < 0.01 are considered significant values, i.e. R^2^ ≥ 0.61.

## Results

**Figure 2:**
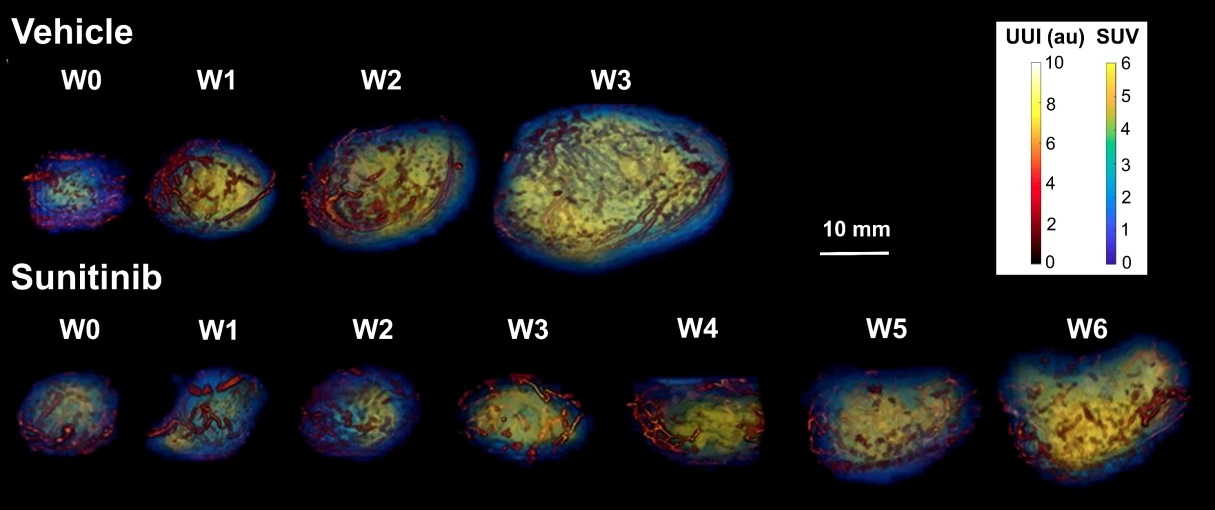
Representative images of tumor vascularization and metabolism acquired weekly in a VEH (top row) and a SUNI-treated mouse tumor (bottom row). Maximum intensity projection (MIP) visualization. Bar Length: 10 mm. Resume of FDG at W3 and growth and vascular network at W5-6 in sunitinib treated animals.

**Figure 3:**
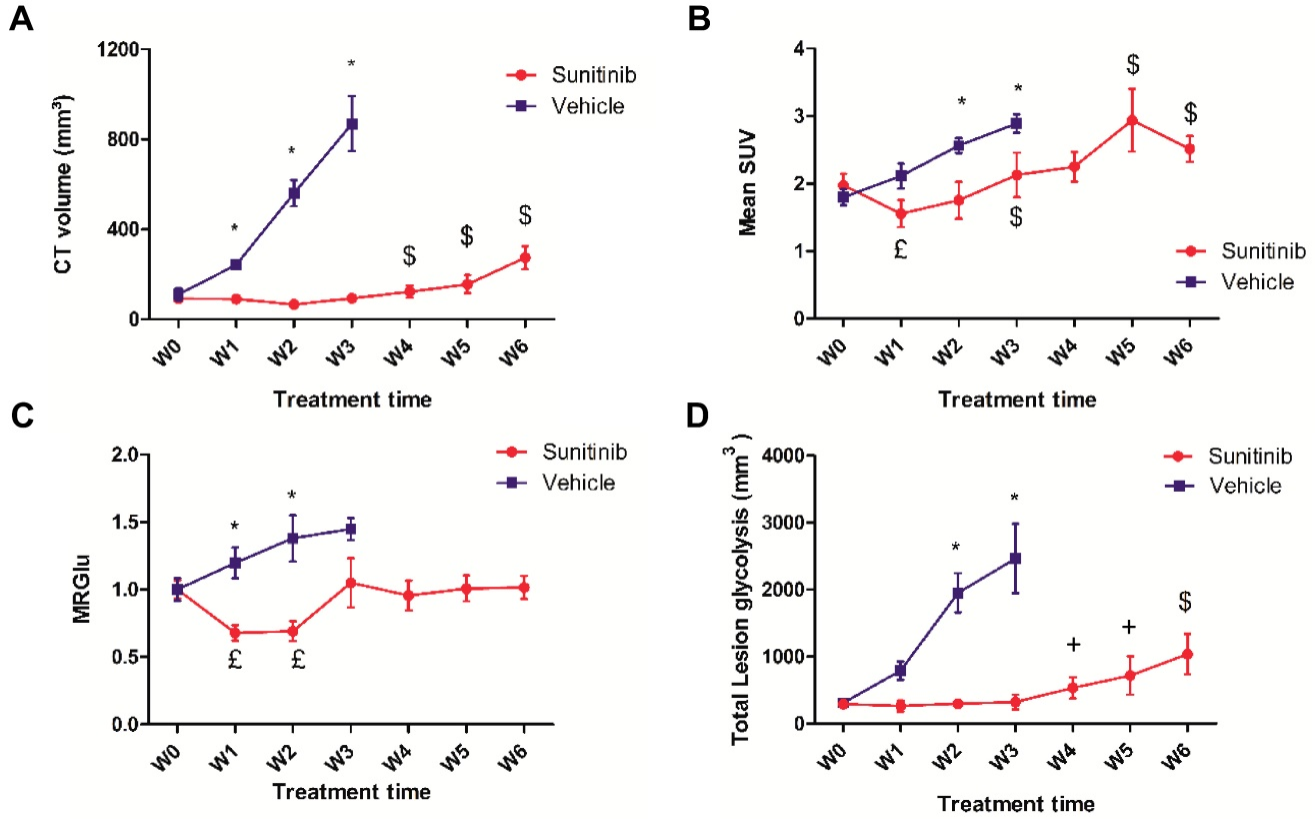
** Effects of sunitinib on tumor growth and glycolytic metabolism of Sdhb^-/-^ PGLs allografts imaged with PETRUS.** Quantification of **(A)** CT volume, ^$^: p <0.05 compared with W0, W1, W2 and W3 in SUNI group. **(B)** Mean SUV by PET, ^£^: p <0.05 compared with baseline in the SUNI group, ^$^: p <0.05 compared with W1 in the SUNI group. **(C)** Metabolic Rate of Glucose (MRGlu): Means were normalized by the baseline value. ^£^: p <0.05 compared to baseline. **(D)** Total Lesion Glycolysis, ^$^: p <0.05 compared to the earlier weeks in the SUNI group; ^+^: p<0.05 in comparison with W0, W1, W2 and W3 in the SUNI group. Data are expressed as mean ± SEM. ^*^: p <0.05 between the two groups with 2-ways ANOVA. In the VEH group, all values at W1 and W3 are statistically different from baseline (p<0.05).

In vivo imaging data of tumor metabolism, anatomy and vascularization were acquired weekly in vehicle (VEH) and sunitinib (SUNI)-treated mice. Results for representative animals are shown in Figure 2. In VEH animals, tumors grew rapidly and reached the maximal tolerable tumor size at week 3 (W3). In contrast, tumor growth stopped in sunitinib-treatment until W3, and at that time tumor volume in SUNI was not different from baseline. Tumor growth then resumed, and at week 6 SUNI tumor volumes were similar to those of week 1 (W1) of VEH (Figure 3A).

### Longitudinal PET shows a transitory effect of sunitinib on FDG uptake

In untreated (VEH) animals, FDG uptake increased with tumor growth. Semi-quantitative (mean SUV: **Figure 3B**) and quantitative (MRGlu: **Figure 3C**) parameters describing glucose metabolism were 30-50% higher at W3 than at baseline, demonstrating that tumor growth was concomitant with a parallel increase in glucose metabolism. However, in sunitinib-treated (SUNI) animals, mean SUV was reduced with respect to baseline at W1, and increased afterwards: at W3, mean SUV had reached pre-treatment baseline values and continued to progress until W5-6 (**Figure 3B**). Thus, the effect of sunitinib on FDG uptake was clearly transitory and, after the first two weeks of treatment, mean SUV increased in SUNI at a rate similar to that of the VEH control group. Quantitative analysis of the same dataset, applying compartmental analysis in order to derive the metabolic rate of glucose utilization (MRGlu) from the dynamic PET scans (see Methods), further confirmed this trend: MRGlu was 30-40% lower at W1 and W2 than at baseline, resumed baseline levels at W3 and remained at this value until W6 (**Figure 3C**). We also calculated another parameter, the total amount of glucose uptake by the tumor (Total lesion glycolysis, TLG, **Figure 3D**) that combines glycolysis and tumor growth and is used clinically. In VEH, TLG raised steadily from baseline until W3, while in SUNI, TLG remained at baseline values during the first 2-3 weeks of treatment, after which it increased again. Taken together, these results show that sunitinib inhibits tumor glycolysis transitorily, even when continued several weeks at clinically-relevant doses.

### The inhibitory action of sunitinib on tumor vascularization is also transitory

**Figure 4:**
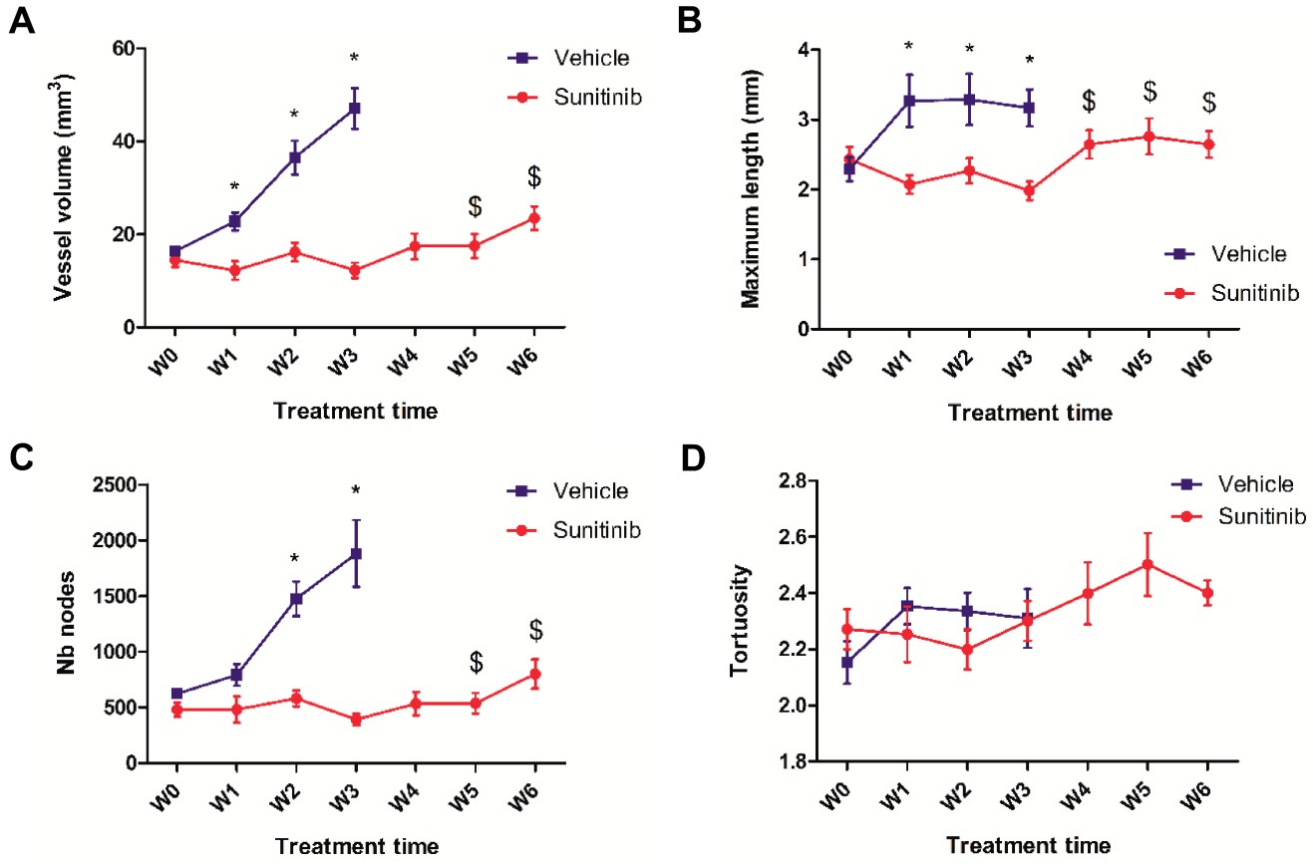
** UUI parameters**. **(A)** Vessel volume, $: p <0.05 compared with W3 and baseline in SUNI group. All VEH values at W1-3 are statistically different from baseline**. (B)** Maximum vessel length. ^$^: p <0.05 in comparison with W3 and W1 in SUNI group**.** Values of VEH are statistically different from baseline at W1 and W2 but not at W3. **(C)** Number of nodes. ^$^: p <0.05 in comparison with W3 and baseline in SUNI group. Values of VEH are statistically different from baseline at W2 and W3. **(D)** Tortuosity. Data expressed in mean ± SEM. ^*^: p <0.05 between the two groups with 2-ways ANOVA.

Simultaneous acquisition of ultrasensitive Doppler allowed to measure several vascularization parameters. In the VEH group, the total vessel volume increased drastically and constantly up to the W3 endpoint when it reached 4 times the baseline value (**Figure 4A**). Similarly, the number of nodes increased up to W3 (**Figure 4C**), while the maximal length and tortuosity (**Figure 4B, D**) increased during the first week and then plateaued. This suggests that the increase in tumor vascularity was largely due to vessel sprouting rather than to the growth of pre-existing vessels.

In the SUNI tumors, there was a small but not significant decrease in vessel volume and maximal vessel length at W1 compared to baseline. Overall, vascularity parameters remained at baseline values until W3-W4, but increased afterwards, at W5-W6. This biphasic time line was different from that of the tumor metabolism: firstly, in contrast to the clear reduction of metabolism, vascularity parameters showed minor fluctuations during the first weeks of treatment. Secondly, the resumption of vascularity occurred later than that of metabolism, suggesting that the latter preceded the former during tumor escape under sunitinib treatment.

Overall, the chronological sequence of events in sunitinib-treated *Sdhb^-/-^* tumors can be summarized as (1) an early (W1) inhibition of tumor growth, metabolic activity and neovessel formation, (2) a resumption of metabolism at W3, followed by (3) a resumption of vascular growth and tumor growth at W4-5.

### Concurrent vascular-metabolic imaging shows that vessels grow preferentially in regions with high glycolytic activity

**Figure 5:**
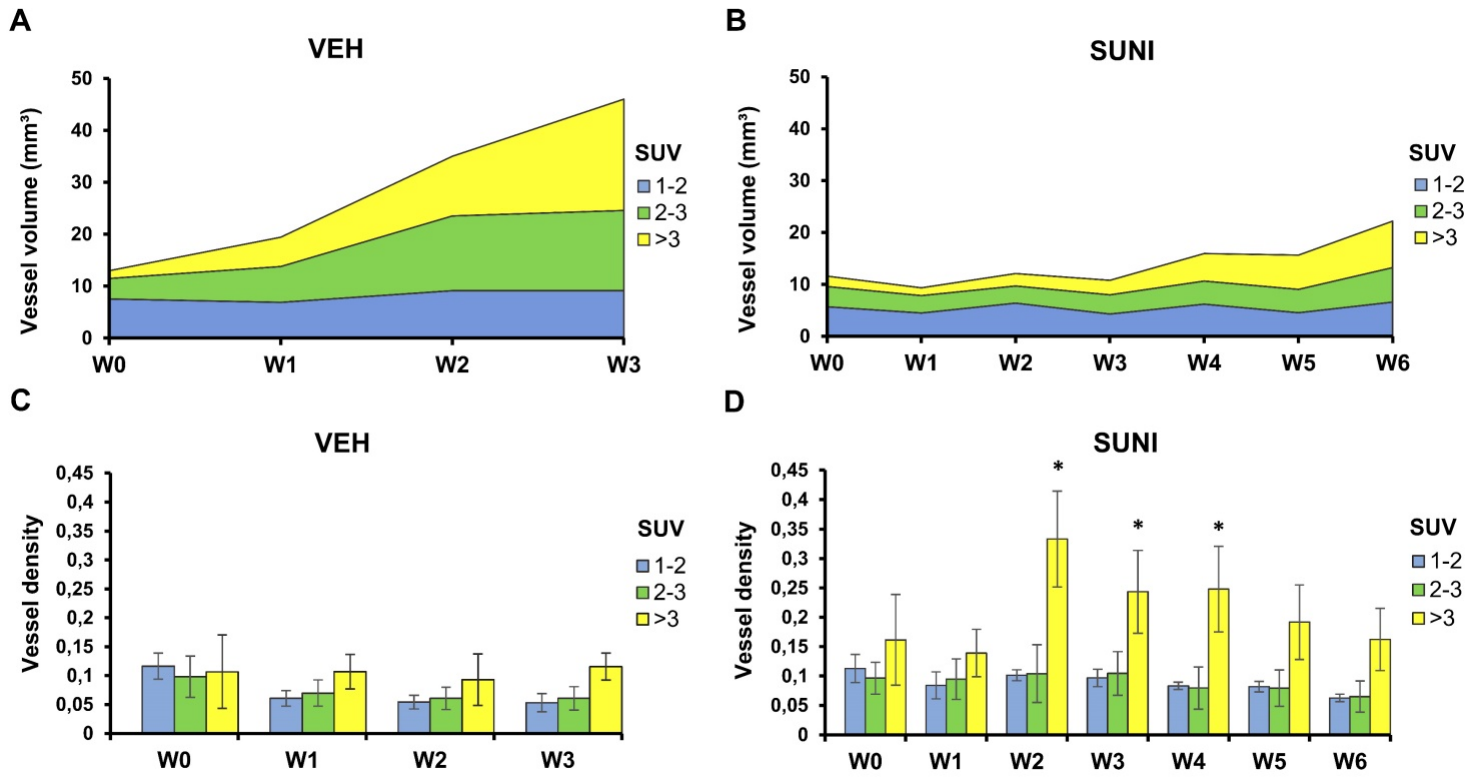
** Perfused vessels feed high metabolic regions during escape to sunitinib**. Each tumor was segmented according to its local mean SUV values into 3 regions: low SUV (1 ≤ mean SUV < 2, blue), intermediate SUV (2 ≤ mean SUV < 3, green), and high SUV (mean SUV ≥ 3, yellow). Panels **A** and **B** present the vessel volume in the segmented regions. Note that these numbers are independent from the relative volumes of each segmented region. Panels** C** and **D** present the ratio of the vessel volume to the volume of each segmented region, which corresponds to the regional vessel densities (vessel volume/ segmented metabolic region). Vessel volumes increase in regions with SUV > 2 in VEH animals (**A**) and, after W4, in SUNI animals (**B**). Minor changes in the vessel densities relative to SUV-segmented regions were observed in VEH animals (**C**). In contrast, in SUNI animals, there was a drastic rise of vessel density in highly glycolytic regions from W2 onwards (**D**). Data are expressed in mean ± SEM. *: p <0.05 between the high SUV-regions and the low or intermediate SUV regions, Wilcoxon matched-pairs test.

We took advantage of the fact that, using PETRUS, PET and UUI are acquired simultaneously and co-registered to the same spatial reference frame. Thus, we segmented each tumor volume into low (mean SUV ≤2) medium (2<mean SUV≤3) and high (mean SUV>3) metabolic regions and measured the vessel volumes separately inside these segments (**Figure 5**). In VEH animals, we observed no change in the vessel volume in regions with low metabolic activity. Vessel volume increased in regions with medium and a high FDG uptake, and the increase was highest in regions with a high metabolic activity (**Figure 5A**). Accordingly, the ratio of vessel volume over tumor volume was roughly similar in the three metabolic segments at baseline (*ca.* 10%), and dropped to *ca*. 5% at W3 in low and medium metabolic regions (**Figure 5C**). This suggests that, as *Sdhb^-/-^* tumors grow in size, they develop vessels in areas with intense glucose metabolism.

In SUNI animals, the vessel volume remained stable in regions of low metabolic activity over the 6 weeks of observation, but, starting at W3-4, increased in regions with medium and, mostly, high metabolism (**Figure 5B**). Remarkably, at week 3, the ratio of vessel volume over tumor volume showed a dramatic increase in high metabolic regions, which reached 30-35% at that time point (**Figure 5D**). Since the total vessel volume remained at baseline levels at this time point, these results indicate that the resumption of metabolism under sunitinib at W3 essentially occurred in the highly-perfused regions.

### Sunitinib treated tumors show a high level of metabolic heterogeneity

**Figure 6:**
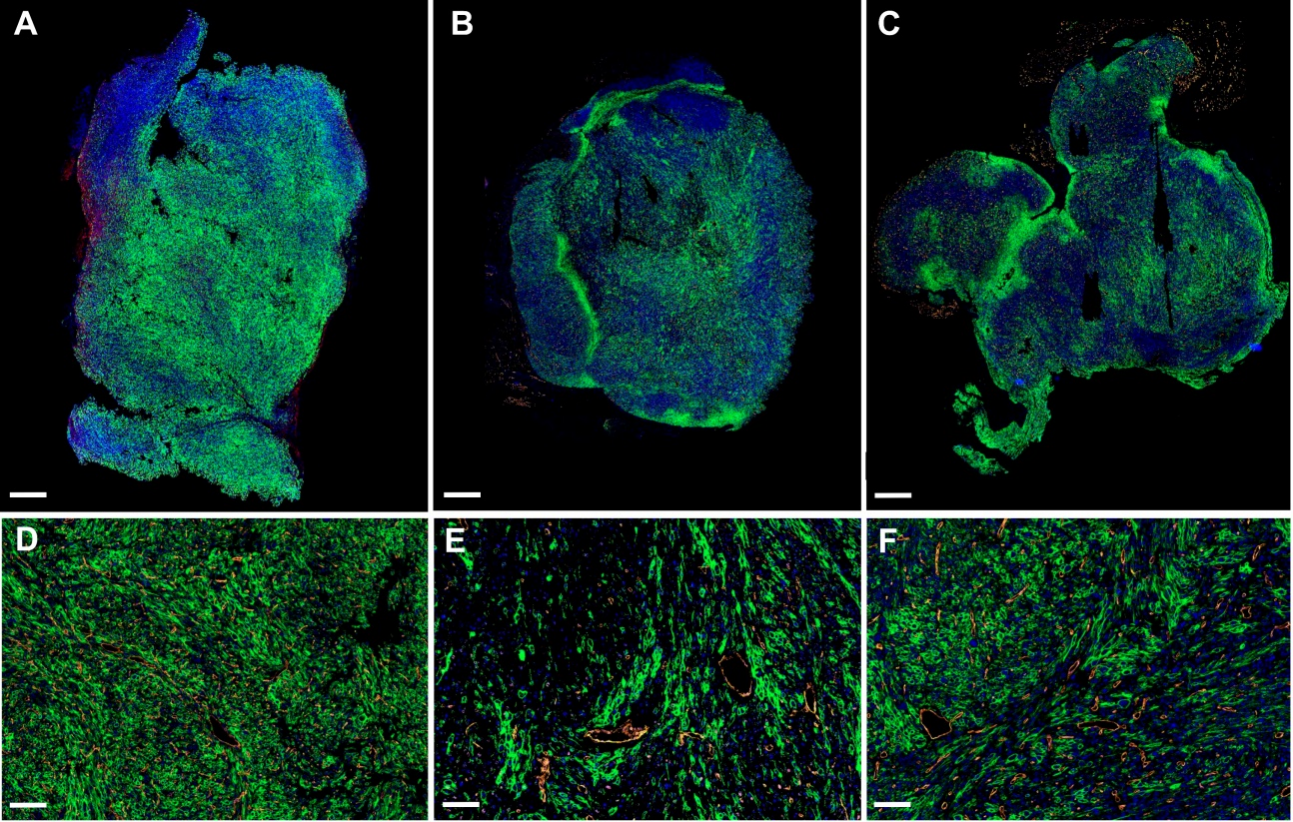
** GLUT1 and CD31 immunofluorescence.** Representative sections of Sdhb^-/-^ tumors at W3 treated with vehicle at W3 **(A, D)**, sunitinib at W3 **(B**, **E)** and at W6 **(C, F)**. Green: GLUT1. Orange: CD31. Blue: DAPI. Length bars: **(A-C)** 800µm, **(D-F)** 200µm.

The propensity of vessels to grow in highly glycolytic regions in the tumors of sunitinib-treated animals prompted us to examine the expression of GLUT1, the major transporter of glucose in SDHB tumors. Surprisingly, the global expression of GLUT1 relatively to tumor weight was not significantly different in the VEH and SUNI animals and was similar at all time points in each group (**Supp. Figure 1**). However, when we examined GLUT1 expression at the microscopic level using immunohistochemistry, we found different patterns of expression throughout the tumors of the VEH and SUNI animals (**Figure 6**). While, in VEH, GLUT1 was homogeneously distributed throughout the tumor (**Figure 6A**), in SUNI we observed a heterogeneous distribution, where patches of high GLUT1 expression were juxtaposed to regions with low or no visible expression (**Figure 6B, C**). Furthermore, we investigated if the regions with the highest GLUT1 expression were more hypoxic. To this end, we quantified by qRT-PCR the expression of VEGF-A in different tumor regions sampled according to their level of GLUT1 immunofluorescence staining (IFS; **Supp. Figure 2A**). At 3 weeks of sunitinib treatment, we observed a 2-fold increase of VEGF-A expression in the tumor regions with high GLUT1 IFS (**Supp. Figure 2A**) with respect to VEGF-A expression in regions with low GLUT1 IFS. Thus, *ex* vivo results were in line with the *in vivo* results shown in **Figure 5.**

Both the histological findings and the sequence of changes that we observed *in vivo*, i.e. resumption of glycolytic metabolism preceding the resumption of vessel growth, raised a question about the causality between metabolic and vascular responses. The fact that a similar sequence was observed in SUNI treated animals led us to infer that the effect of the drug on angiogenesis could be transitory and fade away after a few weeks of treatment. To explore this possibility, we stained tumor tissue sections for CD31 expression in endothelial cells and α-SMA expression in pericytes.

**Figure 7:**
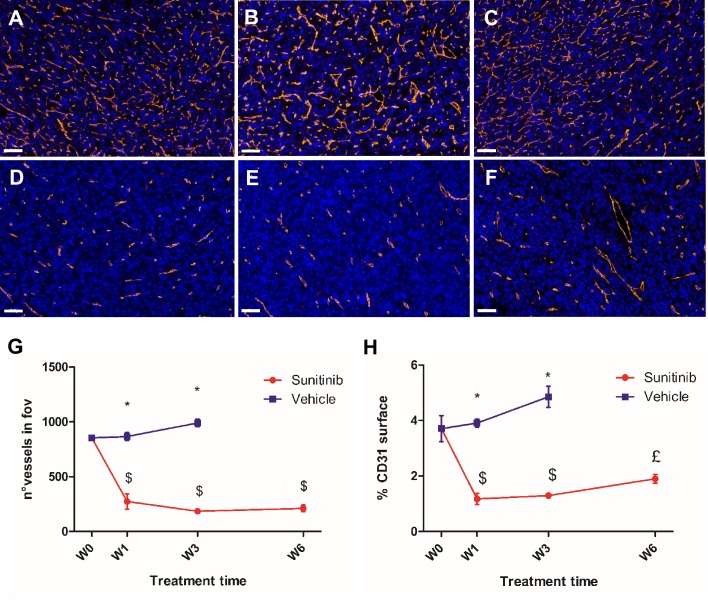
** Sunitinib decreases the microvascular density and the number of vessels.** Representative sections of Sdhb^-/-^ tumor stained for blood vessels (CD31 staining, orange) and nuclei (DAPI, blue). Length bar: 50µm.** (A)** Baseline, **(B)** VEH 1 week, **(C)** VEH 3 weeks, **(D)** SUNI treated 1 week, **(E)** SUNI 3 weeks, **(F)** SUNI 6 weeks. Magnification 20x. **(G)(H)** Quantification of number of vessels and percentage of CD31 surface in field of view in tumors treated with VEH or with SUNI. ^$^: p <0.05 in comparison with the baseline with Student's t-test. ^£^: p <0.05 in comparison with baseline and W3 of SUNI. Data expressed in mean ± SEM. ^*^: p <0.05 between the two groups with 2-ways ANOVA.

CD31-stained vessels in the VEH tumor sections were abundant, tortuous and variable in length (**Figure 7A**-**C**). Interestingly, quantitative analysis of the sections found no change in the number of vessels per field of view during the 3 weeks of tumor growth (**Figure 7G**), though the density of CD31 staining (**Figure 7H**) increased slightly (+15% between baseline and W3, not statistically significant). In contrast, vessels in the SUNI group (**Figure 7D- F**) were dramatically reduced in number (**Figure 7G**) and density (**Figure 7H**). Interestingly, at W3 in VEH and at W6 in SUNI, the CD31 surface parameter increased but not the number of vessels (compare **Figure 7G** and **Figure 7H**), suggesting an increase in vessel diameter.

**Figure 8:**
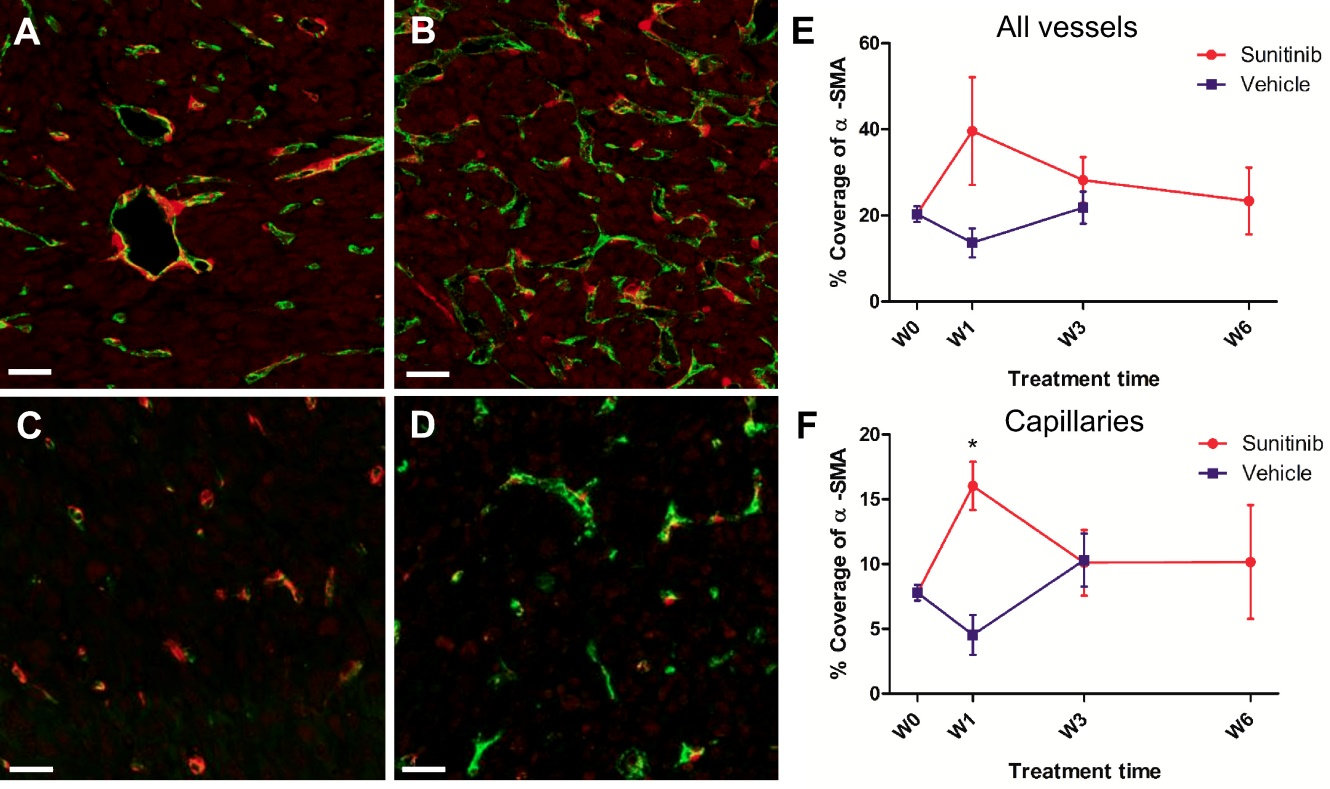
** Pericyte coverage changes during sunitinib treatment.** Representative sections of Sdhb^-/-^ tumor stained for pericytes (α-SMA staining, red) and endothelial cells (CD31 staining, green). **(A)** Week 1 and **(B)** week 3 in VEH tumors. **(C)** Week 1 and **(D)** week 3 in SUNI tumors. Length bars: **(A)-(D)**: 30µm. **(E)** and **(F)** percentage of pericyte (α-SMA) coverage (number of covered vessels/total vessel number) in tumors treated with VEH or with SUNI. **(E)** All vessels, **(F)** only capillaries (diameter <10µm) considered. Data expressed in mean ± SEM. *: p <0.05 between the two groups at W1 with 2-ways ANOVA.

Similarly, double staining of tumor sections for pericytes with α-SMA and endothelial cells with CD31 showed little differences over time in the VEH and SUNI groups (**Figure 8**), except for one noticeable, although not statistically significant, increase of pericytes coverage at W1 in the SUNI tumors (**Figure 8E**, **F**).

Overall, the results clearly indicate that a very low level of CD31 expression was maintained throughout the 6 weeks of sunitinib treatment, while there was a clear increase in perfused vessel volume over the same period.

### The changes in maximum vessel length and total lesion glycolysis are early biomarkers of CT-PET-UUI resistance to sunitinib treatment

**Figure 9:**
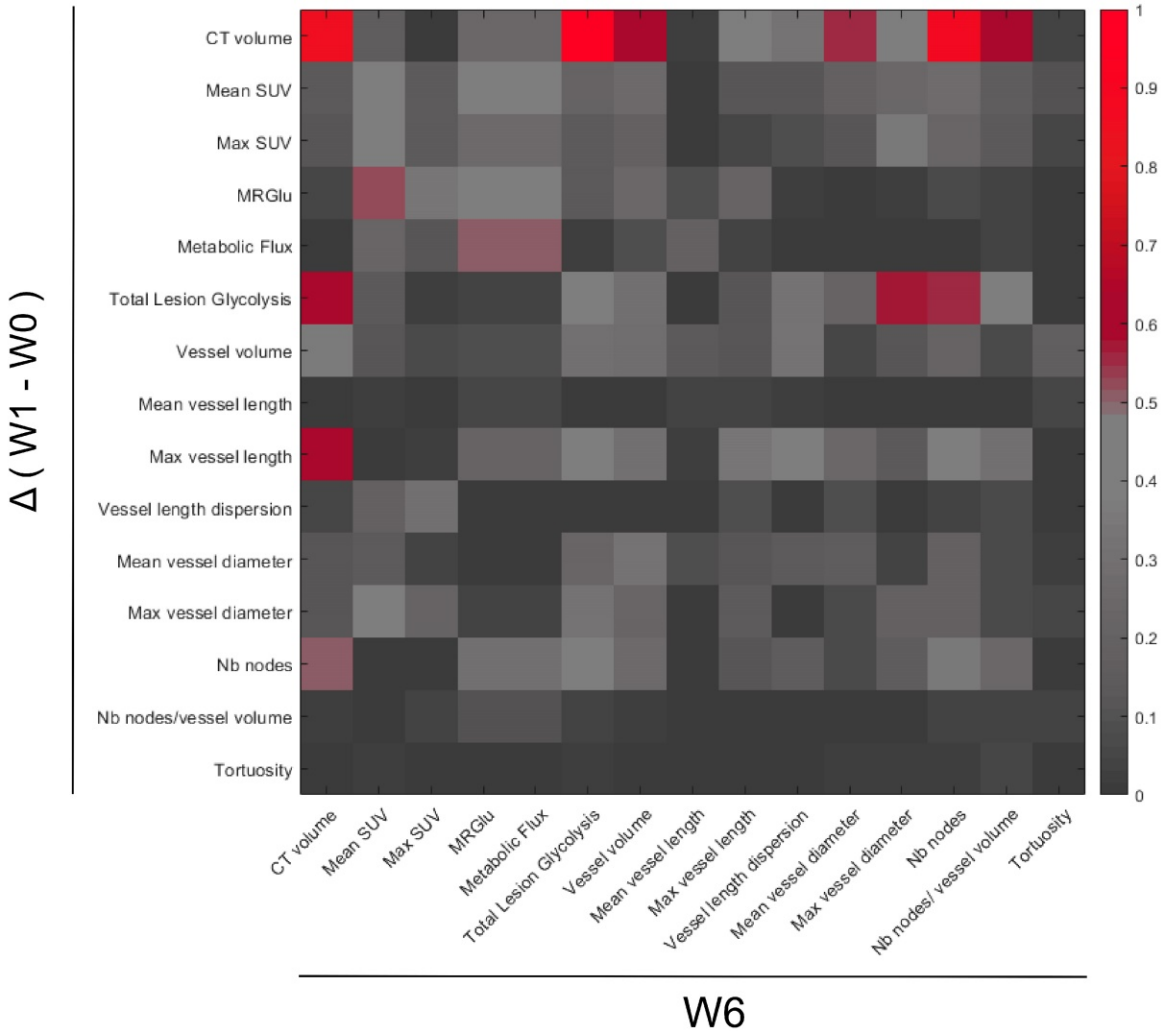
** Correlations between PET-CT-UUI parameters at the week 6 (W6) and the variations between Week 1 (W1) and baseline (W0).** Heatmap plotting the square of Pearson correlation coefficient values (R^2^). Parameters variations between W1-W0 (y-axis) and parameter values at W6 (x-axis). Parameters in the two axis: CT volume, mean and max SUV, MRGlu, Metabolic Flux, Total Lesion Glycolysis, Vessel volume, Mean and Max vessel length, Vessel length dispersion, Mean and Max vessel diameter, Number (Nb) of nodes, Number of nodes/ vessel volume, and Tortuosity.

Searching for an early response biomarker capable of predicting resistance to sunitinib, we calculated the correlations between the changes of all imaged-derived parameters from baseline to W1, and their values at W6 (**Figure 9**). Considering the small size of the sample (n= 12 mice), we fixed the significance level at p=0.01 **(R^2^ ≥ 0.61).**

Regarding the metabolic parameters, changes of mean SUV and maximum SUV after 1 week of treatment were poorly correlated to tumor size at W6 (R= 0.37 and 0.34 respectively). In contrast, the W1 changes in Total Lesion Glycolysis (TLG), a parameter that combines tumor volume with mean FDG uptake, were correlated with tumor size at W6.

Regarding the vascular parameters, the reduction of maximum vessel length at W1 was correlated with the final tumor size at week 6 (R=0.78), indicating that the larger the inhibitory effect of sunitinib on vessel length, the smaller the tumor volume at W6.

## Discussion

Tissue perfusion and metabolism have a direct link: vessels bring to tissues the substrates necessary to sustain metabolic needs. This is of particular importance to tumors, characterized by an uncontrolled growth, a high need for substrates, the uptake of massive amounts of circulating glucose regardless of the oxygen state (Warburg effect), and the formation of an abnormal vascular network through neoangiogenesis and neovasculogenesis.

Imaging techniques capable to follow in parallel tumor hallmarks 38 non-invasively provide exquisite insight into the link between deregulated energy metabolism and neoangiogenesis/vasculogenesis during tumor growth 17. PETRUS is a new preclinical *in vivo* imaging device that can image non-invasively, simultaneously and longitudinally, (i) anatomy with CT, (ii) tumor perfusion using the ultrafast ultrasound Doppler mode, and (iii) glucose metabolism using FDG dynamic PET 18. In the present study, PETRUS measured longitudinally perfusion and metabolism parameters (glucose uptake and consumption) in a mouse model of the human cancer paraganglioma/pheochromocytoma (PPGL) and explored the effects of sunitinib, the reference anti-angiogenic drug under clinical trials for this type of cancer 39. PPGL are frequently inherited tumors with an important genetic heterogeneity, with* SDHB* mutations being the most frequently associated with a metastatic disease. In these patients, loss of succinate dehydrogenase (SDH) function has been associated with succinate accumulation 40, leading to a pseudohypoxic phenotype 41 and therefore to increased anaerobic glycolysis and angiogenesis 42-45. SDH-deficient PPGL patients are thus natural candidates for anti-angiogenic treatment 4. Several clinical trials evaluating the response to sunitinib in patients with malignant PPGL are ongoing (i.e. the FIRSTMAPP randomized phase II European clinical trial) or have recently been published 7,9 . Although sunitinib did show some efficacy, partial response or escape has been described 9,10,39. This resembles the situation in renal cancer, for which sunitinib increases life expectancy of metastatic patients 3 but with a frequent occurrence of acquired resistance after an initial phase of positive response 46,47. Recently, several reports have suggested that sunitinib resistance was linked to a metabolic switch of the tumor towards anaerobic glycolytic metabolism, rather than to insensitivity to VEGF blockage 14-16. However, this link has not been established in *SDHx*-mutated PPGL, in which pseudo-hypoxia interferes with both metabolism and angiogenesis.

Our study reports the sequence of metabolic and vascular events during sunitinib treatment in the *Sdhb^-/-^* tumor mouse model. Immediately after initiation of treatment, sunitinib stops tumor growth and vascular development and decreases glucose metabolism of the tumors. Stabilization or reduction of the quantitative parameters describing these three tumor hallmarks occur after one week of treatment, at a time when the same parameters are significantly increased in the vehicle-treated group. At the macroscopic level *in vivo*, FDG uptake (Mean SUV) and consumption (MRGlu) were reduced at W1, while tumor volume and vascular parameters were identical to pre-treatment values. At the microscopic scale, sunitinib induced a drastic reduction in vascular density along the 6 weeks of treatment. The reason for which PETRUS did not detect early vascular changes under sunitinib treatment is probably due to the spatial resolution of the UUI Doppler images, currently limited to 95µm 18. Using higher resolution UUI of Lewis lung carcinoma (LLC) in mice, Demené *et al.* found 74% of vessels with a diameter of less than 200µm 48. Other authors, using DCE-US 49 or both non-enhanced and contrast-enhanced 3D Doppler 50 showed reduced tumor vessel volume early after the initiation of sunitinib treatment in murine LLC. In its present state of development, PETRUS is blind to small venules, arterioles and capillaries and would miss any effect of sunitinib on tumor capillaries. Nevertheless, PETRUS can clearly document the changes in tumor vessels larger than 95-100 µm.

UUI Doppler in VEH tumors showed that tumor perfusion significantly increased at W1. This raises the question as to the nature of vessels, or neovessels, that support increased perfusion during the growth of PPGL tumors. The present results provide an array of evidence suggesting that increased tumor perfusion during the escape from sunitinib is the consequence of a change in the tumor vessels' morphology. This is objectivized by an increase in total vessel volume, maximal length, number of nodes at 4-5 weeks of sunitinib treatment, i.e. during vascular escape. In contrast, these parameters increased continuously (total vessel volume and number of nodes) or remained at higher-than-baseline levels (maximal length) in vehicle-treated animals. Tumor vessels often have a weak pericyte coverage, which increases leakage by destabilization of adherent junctions in ECs 51. Indeed, an increase in pericyte coverage is typical of the vascular “normalization” observed after a few days of anti-angiogenic therapy, when vessels tend to stabilize their structure and function 52, 53. Here, increased pericyte coverage demonstrated by α-SMA expression, a protein specific of pericytes, was transiently observed at W1 of sunitinib treatment but not later. Taken together, these results show that the increase in tumor perfusion parameters, both in VEH animals and in SUNI animals after 4-5 weeks, was linked to increased blood flow in larger vessels rather than to the formation of CD31-stained vessels. Accordingly, dilatation of tumor vessels and increase in vessel diameter during tumor growth were also reported in an experimental model of glioma in mice using high resolution intravital optical imaging 54.

In agreement with previous clinical studies, sunitinib treatment did not durably stop tumor growth of *SDHB*-deficient PGLs 10. We show here that the escape from metabolic inhibition (W3), indicating an adaptive mechanism of metabolic resistance after 2-3 weeks of sunitinib, precedes the resumption of tumor growth (W4) and vascularization (W4-5). Metabolic adaptation under sunitinib treatment has recently been described in animal models 15,16 and in renal cancer 14. The results from these studies support the “metabolic symbiosis” hypothesis of tumor development raised by Sonveaux and colleagues 55. In brief, metabolic symbiosis refers to tumor heterogeneity, where acute hypoxia causes the clusterization of cancer cells according to their proximity with perfused blood vessels. In hypoxic clusters, cancer cells metabolize glucose anaerobically and secrete lactate, while in normoxic clusters, cancer cells import and metabolize lactate aerobically 55. Sonveaux's hypothesis in tumors is comparable to the demonstration by Magistretti and Pellerin of the astrocyte-neuron metabolic shuttle in the central nervous system, where astrocytes metabolize glucose anaerobically into lactate with which they “feed” aerobic neurons 56.

Our results are partly in agreement with the hypothesis of metabolic symbiosis. Under sunitinib treatment, we observed clusters of GLUT1 at the macroscopic **(Figure 6 A-C)** and microscopic levels **(Figure 6 D-F)**. In contrast, there was no obvious macroscopic or microscopic clustering for GLUT1 in vehicle-treated tumors at any stage of development (**Figure 6 A** and** D**). It is known that the *Sdhb*^-/-^ phenotype creates a succinate-induced pseudo-hypoxic situation with stabilization of HIF-2α 45,57. Here, at W3 of sunitinib treatment, the clusters of high GLUT1 expression also expressed a high level of VEGF-A (**Supp. Figure 2**), the growth factor binding to (sunitinib-inhibited) VEGFR. VEGF-A expression is induced by HIF-1α stabilization. It has been shown that highly glycolytic tumors develop resistance to anti-angiogenic therapy by favoring hypoxic proliferative regions 58, in which both the mitochondrial dysfunction and the reduction in oxygen and nutriment availability trigger the stabilization of HIF-1α 59,60. In the tumor model used here, the *Sdhb*^-/-^ cells are in a succinate-induced pseudohypoxic state, in which there is stabilization of HIF-2α 39, 52, but grow inside PPGL tumors which receive a consistent blood supply. Sunitinib treatment changes their environment into a hypoxia-sustained microenvironment, stabilizing HIF-1α. In other words, sunitinib creates a “real” hypoxia in cells adapted to pseudo-hypoxia. It is tempting to suggest that the pseudo-hypoxic state may induce a special type of vasculogenesis, and that sunitinib is capable to block vessel sprouting and maturation but would fail to prevent this other type of vasculogenesis. This attractive hypothesis, which calls for further experimentations, is consistent with previous studies showing that pseudohypoxia induces specific vascular patterns, such as the growth of long vessels forming arcs in *SDHB*-PPGL 61. It is also consistent with the increase in maximum vessel length that we observed in the untreated VEH animals between W0 to W1 (**Figure 4 B**).

Naturally, one should remain cautious since in our study, *in vivo* Doppler measured the red blood cells flowing in the tissue, while the endothelial cell marker CD31 revealed both functional and non-functional vessels in fixed (un-perfused) tissue sections. Nevertheless, in support of this hypothesis, our results show that the increase in perfusion under sunitinib takes place essentially in tumor regions with high FDG uptake, i.e. high glycolysis. The inhibition of vessel maturation, efficient during the first weeks of treatment, could lead to a metabolic increase of glycolysis, even in highly glycolytic tumors such as SDH-deficient PPGL. Indeed, this metabolic shift does precede the change in vessel morphology, leading to increased tumor perfusion by a mechanism that remains to be uncovered. Whether the vascular shift is a direct or indirect consequence of the metabolic changes remains speculative and will require the exploration of other tumor types by concurrent metabolic and vascular imaging.

Finally, one of the objectives of the present study was to uncover predictive biomarkers of the response to sunitinib therapy. We have shown here that the decrease in the maximum vessel length and total lesion glycolysis, measured after one week of sunitinib, were correlated with the endpoint tumor volume. The real value of these parameters to predict tumor escape from antiangiogenic treatment remains to be demonstrated in future studies in other tumor models and in the clinical setting.

In the future, PETRUS could serve as an imaging instrument to perform extensive analysis of quantitative parameters of experimental tumors with different metabolic backgrounds and under various treatments. Possible applications of this new imaging methodology could include the prediction of the sensitivity of a given tumor type to a targeted treatment or its escape thereof. Moreover, as it describes simultaneously the time course of several hallmarks of the response to treatment, it may help defining new treatment associations, such as addition of a second drug targeting specifically the mechanism of escape from the first drug, e.g. association of antimetabolites with anti-angiogenics.

## Supplementary Material

Supplementary figures.

## Abbreviations

CD31: cluster of differentiation 31
CT: computed tomography
DCE-US: Dynamic contrast-enhanced ultrasonography
EMA: European medicines agency
FDG: 2′ -deoxy-2′ -[^18^F]fluoro-D-glucose
FIRSTMAPP: first international randomized study in malignant progressive pheochromocytoma and paraganglioma
FDA: food and drug administration
FFPE: formalin-fixed paraffin-embedded
GLUT1: glucose transport protein 1
HIFs: hypoxia-inducible factors
IFS: immunofluorescence signal
imCC: immortalized mouse chromaffin cells
LLC: Lewis lung carcinoma
Max SUV: maximum SUV
MRGlu: metabolic rates of glucose
PET: positron emission tomography
PETRUS: PET registered ultrafast sonography
PGL: paraganglioma
PPGL: paragangliomas and pheochromocytoma
SDHB: succinate dehydrogenase subunit B
SNIPP: study of sunitinib in patients with recurrent paraganglioma/pheochromocytoma
SUNI: sunitinib
SUV: standard uptake value
TKI: multi-tyrosine kinase inhibitor
TLG: total lesion glycolysis
UUI: ultrafast ultrasound imaging
VEGF: vascular endothelial growth factor
VEGFRs: VEGF receptors
VEH: vehicle
VOI: volume of interest
W0-6: week 0-6
α-SMA: alpha-smooth muscle actin
