# Supplementary material for: Concurrent imaging of vascularization and metabolism in a mouse model of paraganglioma under anti-angiogenic treatment
Source: Theranostics. 2020 Feb 10;10(8):3518–32. doi: 10.7150/thno.40687 (PMC7069082; doi:10.7150/thno.40687)
Supplement: Supplementary file 1 — Supplementary figures. [file thnov10p3518s1.pdf]

Supplementary Figures:

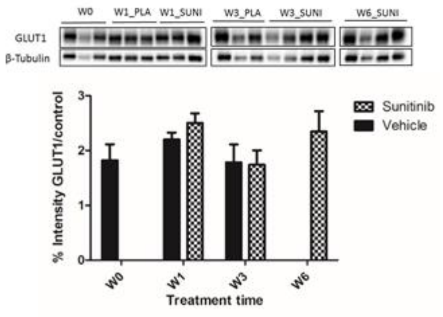

Supplementary Figure 1. Western blots show no difference in GLUT1 expression in SUNI (gray columns) and VEH-treated mice (black columns). Data are duplicate assays of n= 3-4 animals, expressed as mean ± SEM of the GLUT1 band density normalized by the band density of β-tubulin.

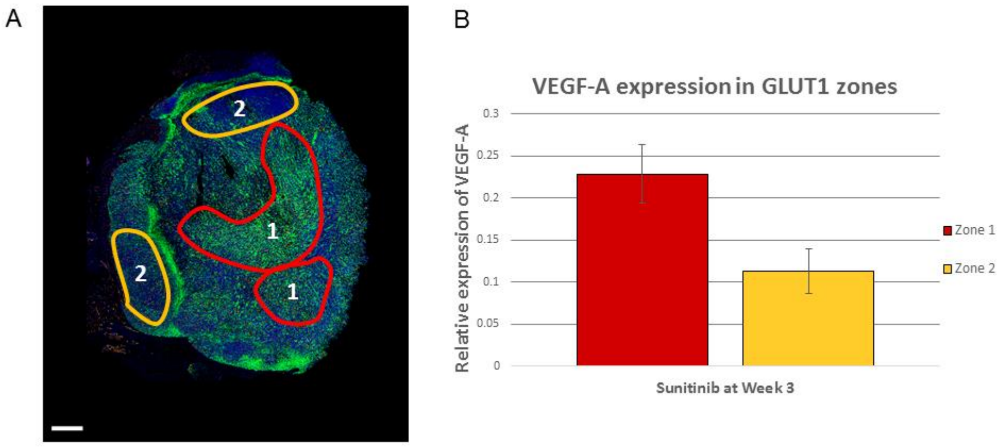

Supplementary Figure 2. Expression of VEGF-A in the GLUT1 regions at week 3 of sunitinib treatment. (A) A representative GLUT1-stained section showing the selection of tumor regions with high GLUT1 expression (zone 1, circled in red) and low GLUT1 expression (zone 2, circled in yellow). (B) Relative expression of VEGF-A in the GLUT1-stained zones 1 and 2. Data are expressed as mean ± SEM (n=5).
